# Investigation of *in vitro* bioactivity, and osteoblast and angiogenic activity of spray-dried boron-doped 58S bioactive glass microspheres

**DOI:** 10.1039/d3ra07472b

**Published:** 2023-12-12

**Authors:** Frizka Vietanti, Tzu-An Lee, Hsiu-Yang Tseng, Yu-Jen Chou

**Affiliations:** a Department of Mechanical Engineering, National Taiwan University of Science and Technology No. 43, Sec. 4, Keelung Road Taipei 10607 Taiwan yu-jen.chou@mail.ntust.edu.tw +886-2-2737-6492

## Abstract

Bioactive glass is a potential biomaterial for bone reconstruction owing to its superior bioactivity and non-toxicity. Yet, the absence of a circulatory system to carry waste and nutrients is a key issue with biomaterials implanted in the body. Thus the development of functional and vascularized new tissue requires the development of angiogenesis, which involves the formation of new blood vessels. Based on this perspective, we aimed to fabricate boron-doped 58S bioactive glass microspheres using the spray drying method, which could offer great flowability, controllable morphology, and narrow size distribution. Characterization of particle morphology and elemental composition were examined using scanning electron microscopy along with energy dispersive spectroscopy, respectively. To evaluate the effect of the boron dopant on *in vitro* bioactivity, X-ray diffraction and Fourier transform infrared spectroscopy were employed, while MC3T3-E1 osteoblast cells and BAOEC endothelial cells were used to assess the *in vitro* osteoblast and angiogenic activities, respectively. Finally, the results showed that two distinct morphologies, smooth and concave spheres, were found, with discussion of the corresponding formation mechanism. In addition, positive effects of the boron dopant were demonstrated on the *in vitro* bioactivity, and osteoblast and angiogenic activity when compared to the un-doped BG specimen.

## Abstract 2

Bioactive glass is a potential biomaterial for bone reconstruction owing to its superior bioactivity and non-toxicity. In this study, spray-dried B-doped bioactive glass microspheres were fabricated, and enhanced *in vitro* bioactivity, osteoblast activity, and angiogenic activity were demonstrated.

## Graphical abstract

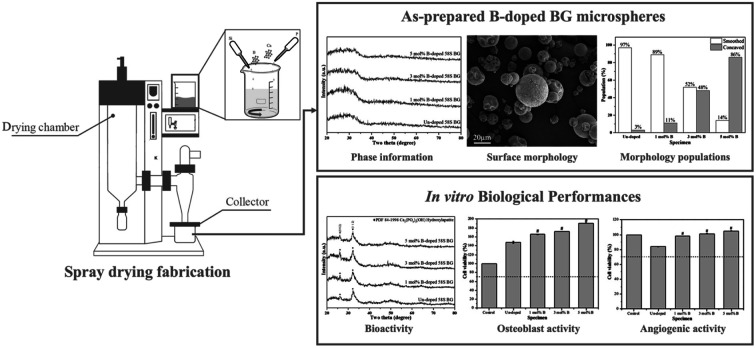

## 1. Introduction

The potential of silica-based bioactive glasses (BGs) to promote osseointegration between implant material and living bone tissues has made them attractive for use in bone implants, due to their outstanding properties including bioactivity, non-toxicity, biocompatibility, osteoconductivity, and biodegradability.^1^ Early research showed that upon implantation within the human body, BGs had the capability to trigger the mineralization of a hydroxyapatite (HA) layer.^2–4^ This process facilitates a chemical bond with human bones, leading to a decrease in inflammation and the potential for rejection.^5,6^ Additionally, among the variations of BG, the composition known as 58S stands out as particularly favorable. This composition possesses notable attributes, such as a reduced tendency for crystallization while maintaining a great conversion rate into HA.^7^

However, a major problem with BG implanted in the body is the absence of a circulatory system to transmit waste and nutrients. Angiogenesis, as the development of new blood vessels, is essential for the formation of new functional and vascularized tissue.^8,9^ Thus, to enhance the biological activity, metallic ions were introduced, which could modify the dissolution behavior and the ionic transformation when exposed to biological fluids. In the past decades, studies have demonstrated that ions such as Mg,^10,11^ Sr,^12,13^ Cu,^14^ and B^15,16^ could improve osteoblast activity and stimulate angiogenesis. Boron, in particular, has been widely shown to induce angiogenesis in both *in vitro* and *in vivo* investigations, which could be greatly utilized for soft tissue repair applications that require vascularization.^17^ In addition, the incorporation of B ions into different proportions of BGs has shown significant effects on bioactivity and osteoblast activity.^18^

To date, the sol–gel method stands as one of the prevailing techniques used for the preparation of B-doped BG. For instance, Rad *et al.*^19^ synthesized the B-doped BG nanoparticles *via* the sol–gel method and evaluated their biological interactions with human dental pulp stem cells. Their study demonstrated that the increase of B dopant in the BG structure could result in reductions in the specific surface areas, pore diameters, and total pore volumes of nanoparticles. In addition, higher B concentrations could enhance early-stage odontogenic differentiation and show higher intracellular calcium levels, indicating its potential in regenerative dental tissue engineering. Meanwhile, Ege *et al.*^20^ prepared sol–gel derived B-containing mesoporous bioactive glasses (MBG) to target muscle regeneration. The resulting MBGs were capable of inducing C2C12 cells to differentiate into myotubes at lower concentrations, suggesting their potential for application in muscle tissue repair. However, disadvantages such as complex procedures, time-consuming steps, and the lack of readily available metal ion precursors have made the sol–gel method only suitable for small-scale production.^21,22^ To overcome these limitations, the spray drying method has advantages attributed to its good flowability, precise size distribution; and controllable morphology, size, and shape, and all these benefits could be achieved through a rapid kinetic process.^23,24^

Therefore, the goal of this work is to prepare the spray-dried microspheres of 58S BG and investigate the biological interactions of the incorporated B dopant. X-ray diffraction (XRD), scanning electron microscopy (SEM), and energy dispersive spectroscopy (EDS) were employed for phase, particle shape, elemental composition, and ion distribution analysis. Further, the *in vitro* bioactivity was assessed *via* XRD and Fourier transform infrared (FT-IR), and the *in vitro* osteoblast and angiogenic activities were evaluated *via* the cell viability of MC3T3-E1 osteoblast cells and BAOEC endothelial cells.

## 2. Materials and methods

### 2.1 Synthesis

In this work, four specimens of 0 mol% (un-doped), 1 mol%, 3 mol%, and 5 mol% B-doped 58S BG were prepared. The 58S BG specimen is constituted of 60 mol% SiO_2_, 35 mol% CaO, and 5 mol% P_2_O_5_. To start with, the precursor solutions were prepared by adding 238.09 g of tetraethyl orthosilicate (Si(OC_2_H_5_)_4_, 99.9%, Showa, Japan), 157.43 g of calcium nitrate tetrahydrate (Ca(NO_3_)_2_·4H_2_O, 98.5%, Showa, Japan), 34.70 g of triethyl phosphate ((C_2_H_5_)_3_PO_4_, 99.0%, Alfa Aesar, UK), and varied quantities (0, 1, 3, and 5 mol%) of boric acid (H_3_BO_3_, 99.5%, Showa, Japan) into 506.97 ml of ethanol. Note that the pH value of each solution was maintained at 2.5 by adjusting with 0.5 M HCl. After stirring for 24 h till homogeneous, the precursor solution was directed into a spray dryer (SDDO-03, IDTA Machinery Co. Ltd, Taiwan). With parameters of input temperature at 200 °C, output temperature at 80 °C, and a 20 ml min^−1^ liquid flow rate. Finally, the dried powders were baked for 12 h at 70 °C and calcined at 600 °C for 1 h (heating rate of 5 °C min^−1^) to receive the final product.

### 2.2 Characterization

First, an X-ray diffractometer (D2 Phaser, Bruker, Germany) was employed to analyze the phase of all 58S BG specimens. The D2 Phaser was equipped with Cu-Kα radiation (*λ* = 1.54 Å), and the XRD patterns were captured at a scanning speed of 0.5 s per step, ranging from 20° to 80°. Next, a scanning electron microscope (6500F, JEOL, Japan) with EDS attachment was used for the characterization of the surface morphologies, elemental composition, and ion distribution. The SEM samples were prepared by distributing the powders onto SEM stubs with conductive carbon tape, which were then coated with platinum. The samples were imaged using secondary electrons at accelerating voltages of 5 kV and a magnification of 1000×. Further, particle sizes and morphology populations were computed by sampling more than 300 particles from the SEM images.

To assess the *in vitro* bioactivity, all 58S BG specimens were immersed into as-prepared simulated body fluid (SBF), following the ISO 23317 protocol.^25^ To begin with, 0.4 g of each 58S BG specimen was soaked in 20 ml of SBF before transferring to an orbital shaker incubator (S300R, Firstek Scientific, Taiwan) and held at 37 °C for 7 d. Note that a daily refresh of the SBF solution was required throughout the examination to simulate the human metabolism system. After the immersion duration, the resulting specimen was carefully rinsed with both de-ionized water and acetone to avoid the formation of salt crystals on the surface. Subsequently, all specimens were dried for a day at 70 °C. To evaluate the formation of hydroxyapatite (HA), both XRD and FT-IR (FTS-1000, Digilab, United States) were employed.

Finally, both *in vitro* osteoblast and angiogenic activity of all 58S BG microspheres were evaluated using the cell viability *via* the 3-(4,5-dimethylthiazol-2-yl)–2,5-diphenyltetrazolium bromide (MTT) assay following the ISO 10993-5 protocol. The MC3T3-E1 osteoblast cells were used to study the osteoblast activity, while the BAOEC endothelial cells were used to examine the angiogenic activity. Initially, the cells were cultured at 37 °C in modified eagle mediums (MEM, Gibco, Massachusetts, USA) with 1 vol% antibiotic (Corning, New York, USA). At a 2 × 10^4^ cells per ml density, the cells were seeded into 24-well plates and cultivated for 1 d at 37 °C in a humidified environment containing 5% CO_2_. Then the BG specimens were put into the well and incubated for another 3 d under identical conditions. Subsequently, 200 μl of MTT solutions were supplemented into each test well, with addition of 300 μl of dimethyl sulfoxide (DMSO) to dissolve the formed formazan. To evaluate the cell viability, a microplate reader (Multiskan Go, Thermo Scientific, USA) was used for the absorption measurements at a wavelength of 570 nm with assessment on a triplicate basis.

## 3. Results

**Fig. 1:**
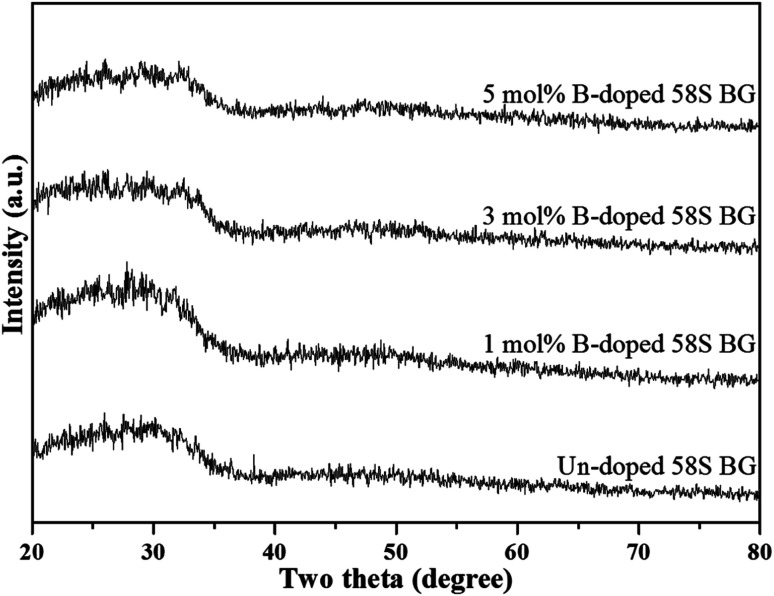
XRD patterns of spray-dried un-doped, 1 mol%, 3 mol%, and 5 mol% B-doped 58S BG microspheres.

To start with, Fig. 1 shows the XRD patterns of all as-prepared spray-dried 58S BG microspheres, with the non-dopant, and 1, 3, 5 mol% B-dopant. According to the graph, a large reflection between 20° to 40° could be observed from the un-doped specimen. This reflection demonstrated that the as-prepared 58S BG microspheres have amorphous phase compositions. Further, for the B-doped specimens, there were no noticeable differences in the XRD patterns as compared to the un-doped BG. This observation suggested that the incorporation of B does not influence the phase composition of the 58S BG microspheres.

**Fig. 2:**
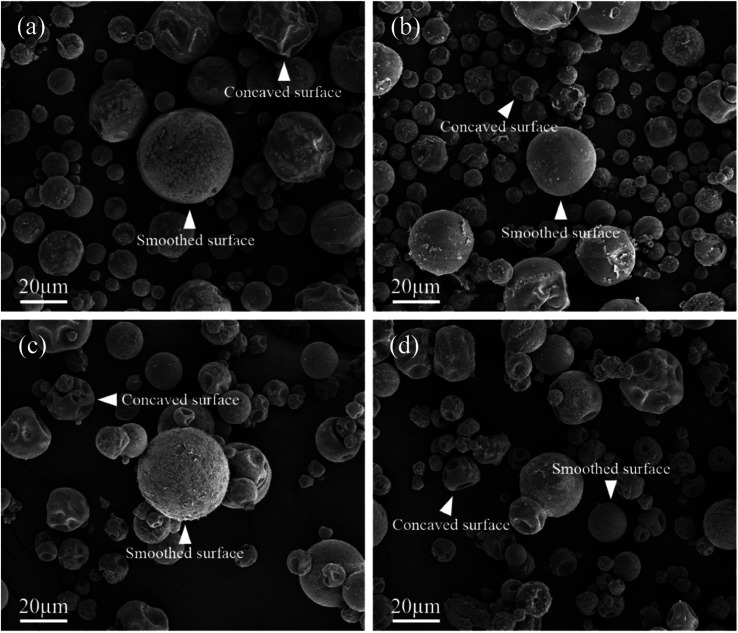
SEM images of spray-dried (a) un-doped, (b) 1 mol%, (c) 3 mol%, and (d) 5 mol% B-doped 58S BG microspheres.

**Fig. 3:**
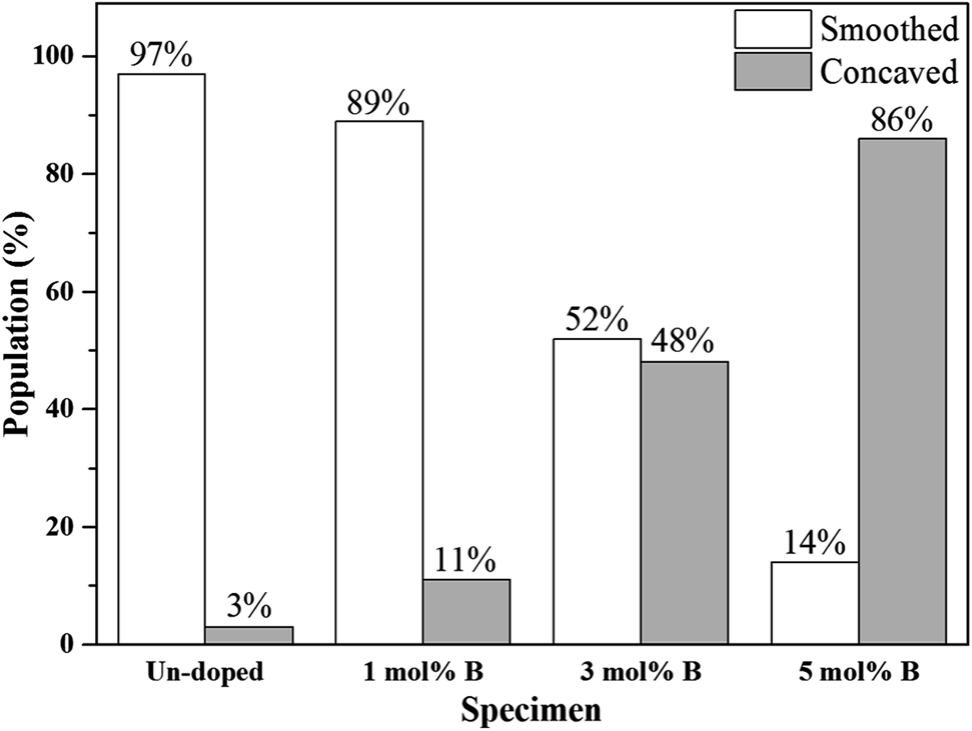
Morphology populations of spray-dried un-doped, 1 mol%, 3 mol%, and 5 mol% B-doped 58S BG microspheres.

**Fig. 4:**
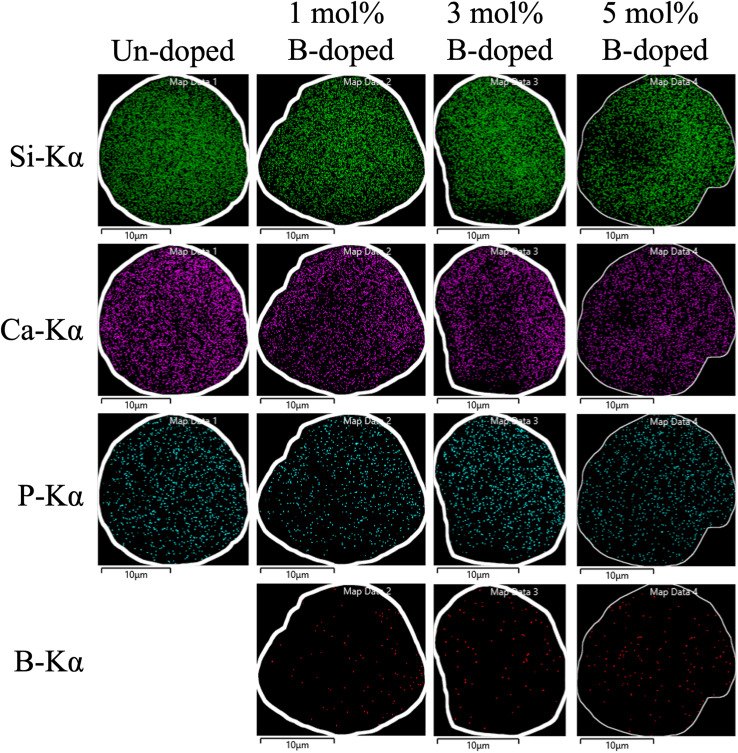
EDS mappings and corresponding Si-Kα, Ca-Kα, P-Kα, and B-Kα distributions of spray-dried un-doped, 1 mol%, 3 mol%, and 5 mol% B-doped 58S BG microspheres.

Next, the SEM images of un-doped and B-doped 58S BG microspheres are presented in Fig. 2. Initially, the average particle sizes of 10.00 ± 5.72, 9.83 ± 6.04, 10.52 ± 6.07, and 9.91 ± 5.26 μm were computed for the un-doped, and 1, 3, and 5 mol% B-doped 58S BG specimens, respectively, showing no difference from un-doped and B-doped specimens. Additionally, it could be seen from Fig. 2(a) that two surface morphologies, smoothed and concaved spheres, were spotted from the un-doped 58S BG specimen. Moreover, similar morphologies (as presented in Fig. 2(b), (c), and (d)) could be identified in the 1, 3, and 5 mol% B-doped 58S BG microspheres. Furthermore, statistical measurements of the morphological populations of all BG specimens are shown in Fig. 3. The results reveal that the un-doped 58S BG microsphere exhibits a smoothed spheres dominated form, consisting of 97% smoothed spheres and 3% concaved spheres (Fig. 3(a)). Meanwhile, for the 1, 3, and 5 mol% B-doped 58S BG microspheres, the populations of smoothed spheres were decreased to 89%, 52%, and 14%, respectively, as shown in Fig. 3(b), (c), and (d). These results show that the quantity of smoothed spheres reduces as the concentration of B dopant increases. Additionally, the elemental compositions and ion distributions of un-doped, and 1, 3, 5 mol% B-doped 58S BG specimens were determined through EDS spectra. Since B cannot be accurately detected by EDX due to low Z elements,^26,27^ the relative content of Si, Ca, and P was investigated regarding the 58S matrix which is presented in Table 1. Further, by utilizing the EDS mappings, the mapping results of all 58S BG microspheres are shown in Fig. 4. The graph shows that the un-doped 58S BG specimen has homogeneous distribution of Si-Kα, Ca-Kα, and P-Kα. Meanwhile, homogeneous distributions could also be observed in the 1, 3, and 5 mol% B-doped 58S BG specimens, which included corresponding B-Kα, demonstrating that B was successfully doped into the 58S BG microspheres.

**Table 1:** Atomic compositions of spray-dried un-doped, 1 mol%, 3 mol%, and 5 mol% B-doped 58S BG microspheres

Specimen	Element concentration (unit: at%)		
	Si	Ca	P
Un-doped	51.55 ± 0.48	40.91 ± 0.94	7.52 ± 1.15
1 mol% B-doped	52.44 ± 2.55	40.20 ± 1.09	7.35 ± 1.81
3 mol% B-doped	52.13 ± 1.39	39.98 ± 1.06	7.88 ± 2.01
5 mol% B-doped	51.71 ± 1.07	40.92 ± 1.78	7.35 ± 0.74

**Fig. 5:**
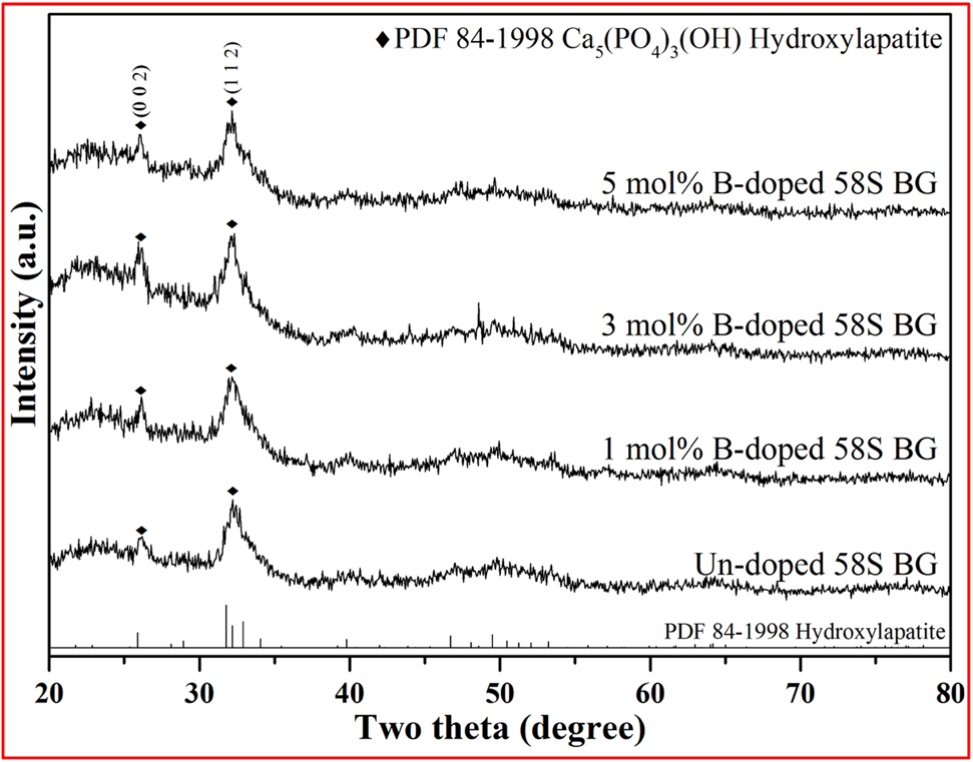
XRD patterns of spray-dried un-doped, 1 mol%, 3 mol%, and 5 mol% B-doped 58S BG microspheres after soaking in SBF for 7 d.

**Fig. 6:**
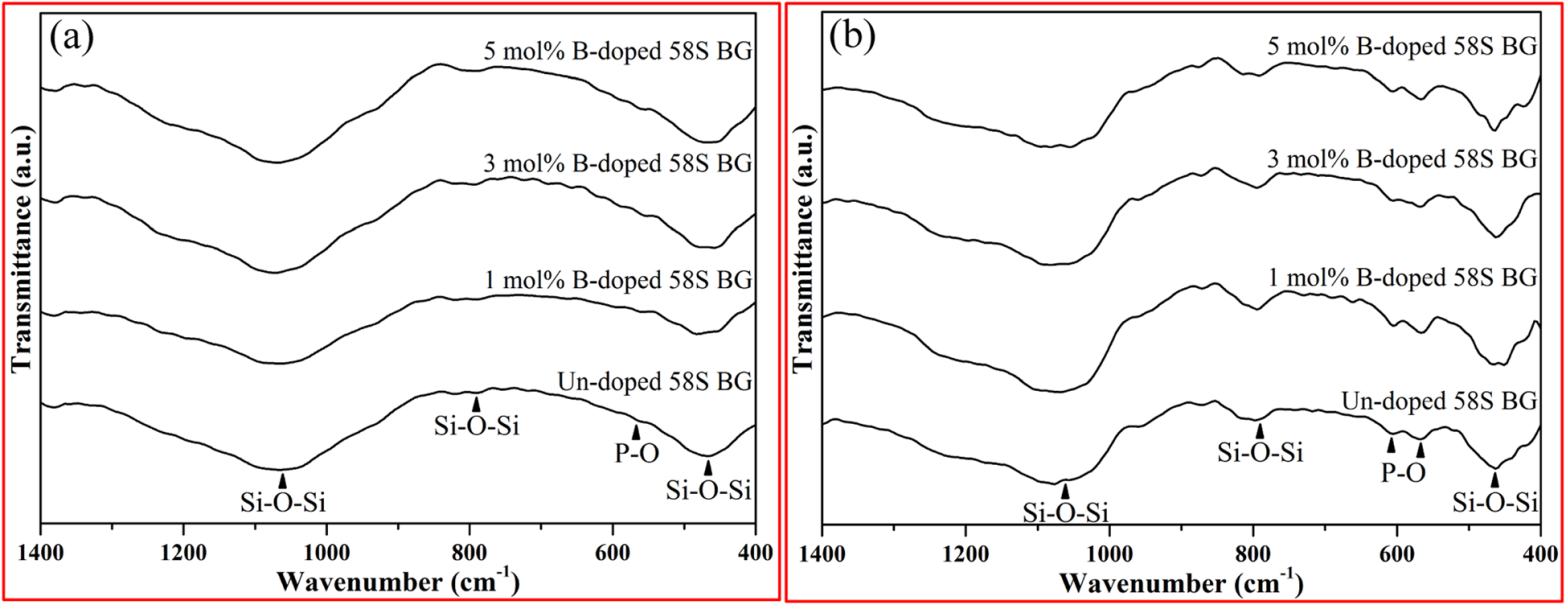
FTIR spectra of spray-dried un-doped, 1 mol%, 3 mol%, and 5 mol% B-doped 58S BG microspheres (a) before and (b) after soaking in SBF for 7 d.

For the assessment of *in vitro* bioactivity, both XRD and FT-IR were employed for the observation of the HA growth after SBF immersion. To start with, the resulting XRD patterns of all SBF-immersed BG specimens are shown in Fig. 5. The data showed that two reflection angles at 26° and 32°, which correspond to the HA crystal (identified through JCPDS 84-1998), could be observed from all BG specimens. Further, to quantify the bioactivity, the FT-IR spectra, as shown in Fig. 6, of all 58S BG specimens were recorded before and after the SBF immersion. According to previous studies,^28^ peaks corresponding to P–O (denoted as *I*_1_) and Si–O–Si (denoted as *I*_2_) bending vibrations could be found at 556 and 482 cm^−1^, respectively. The quantity of formed HA can be assessed by computing the *I*_1_/*I*_2_ intensity ratio. An increased formation of HA leads to a higher P–O bond intensity (*I*_1_), whereas the Si–O–Si base remains unchanged, thereby resulting in a higher *I*_1_/*I*_2_ ratio. In this work, the intensity ratio of the un-doped, and 1, 3, 5 mol% B-doped 58S BG specimens were computed as 0.33 ± 0.09, 0.38 ± 0.05, 0.37 ± 0.04, and 0.35 ± 0.07, respectively, showing the order of bioactivity is 1 mol% B-doped 58S BG > 3 mol% B-doped 58S BG > 5 mol% B-doped 58S BG > un-doped 58S BG. To summarize, both XRD and FT-IR analyses prove the *in vitro* bioactivity of all BG specimens following an immersion in SBF for 7 d.

**Fig. 7:**
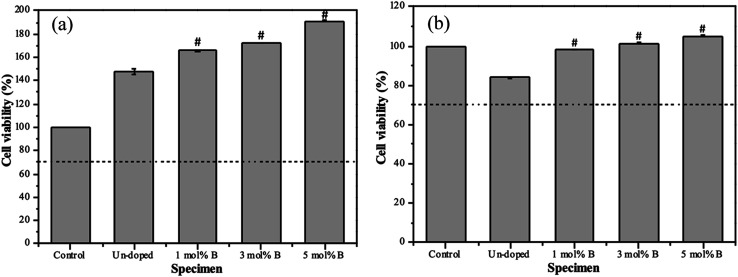
Cell viability of spray-dried un-doped, 1 mol%, 3 mol%, and 5 mol% B-doped 58S BG microspheres derived from (a) MC3T3-E1 osteoblast cells and (b) BAOEC endothelial cells after incubation for 3 d. (#: *p*-value < 0.05).

At last, the MTT assay was used to evaluate the cell viability of all BG specimens, and the osteoblast and angiogenic activities of all specimens are shown in Fig. 7. Initially, for the osteoblast activity (shown in Fig. 7(a)), the cell viability from the MC3T3-E1 osteoblast cells of un-doped and 1, 3, 5 mol% B-doped 58S BG specimens could be computed as 148.3 ± 2.4%, 166.5 ± 0.6%, 172.3 ± 0.3%, and 191.2 ± 0.3%, respectively. These results showed values exceeding the ISO standard threshold of 70%, indicating the non-toxicity of all BG specimens to MC3T3-E1 cells. Additionally, the order of osteoblast activity is as follows: 5 mol% B-doped 58S BG > 3 mol% B-doped 58S BG > 1 mol% B-doped 58S BG > un-doped 58S BG. This order shows a growth in cell viability corresponding to the increased B concentration. Next, for the angiogenic activity (shown in Fig. 7(b)), the cell viability against BAOEC endothelial cells of un-doped, and 1, 3, 5 mol% B-doped 58S BG specimens were measured as 83.9 ± 0.5%, 98.0 ± 0.1, 101.5 ± 0.2, and 105.1 ± 0.1%, respectively. All values are greater than the ISO standard threshold of 70% as well, demonstrating that all BG specimens are angiogenic to BAOEC endothelial cells. Furthermore, an increased amount of B dopant showed a higher angiogenic effect on the lifespan of the endothelial cells. It is worth mentioning that *p*-values were calculated in comparison to the un-doped 58S BG specimen, and all values derived from the B-doped 58S BG microspheres were found to be below 0.05 (indicated as # within the graph), suggesting a significant difference in both osteoblast and angiogenic activities with B dopant.

## 4. Discussion

To start with, the phase information of the 58S BG specimens was discussed. From the XRD data presented in Fig. 1, a broad reflection without any distinct crystalline peaks could be observed, indicating that the spray-dried un-doped 58S BG specimen exhibits an amorphous phase, which agrees well with our previous studies.^29,30^ Furthermore, similar XRD patterns were observed from the B-doped 58S BG specimens. In addition, similar results were reported by Rad *et al.*^19^ with the sol–gel derived B-doped BG, showing that with the increase of B_2_O_3_ concentration of up to 21%, the structure of BG still remains amorphous. Meanwhile, Ege *et al.*^20^ also demonstrated that the structure of MBG after B incorporation of up to 18% remained amorphous. These studies show a good agreement with our work and suggest that the dopant of B into the BG network was successful and has no impact on the phase of the BG structure.

Then, we discussed the particle morphologies of the 58S BG specimens. It could be seen from Fig. 2 that two surface morphologies, smoothed spheres and concaved spheres, were found from the SEM images, along with their statistical morphology populations shown in Fig. 3. The results show that as the B concentration increases, the population of smoothed spheres diminishes while the population of concave morphology increases. Similar to our previous work,^30^ the rate of evaporation during the spray drying process is the primary factor affecting such changes in particle morphology, which is described *via* the Peclet number. In this work, a low Peclet number led to the drying of a molecule with a high diffusion coefficient, thus forming the smoothed spheres. However, with the addition of B, the evaporation rate of the solution was altered and resulted in a higher Peclet number. It then led to surface enrichment and surface precipitation of each droplet and formed the shell-like structure, thus resulting in the morphology of concaved spheres.

For the *in vitro* bioactivity, the spray-dried 58S BG microspheres were examined through SBF immersion. The XRD patterns (Fig. 5) revealed that both un-doped and B-doped 58S BG specimens were confirmed to be bioactive with the identification of HA peaks. In addition, the HA formation could be affected by various factors such as dissolution,^31^ ion exchange,^32^ and crystalline growth^33^ during the SBF immersion, studies have demonstrated that the addition of B within the BG structure could alter the amorphous structure and result in a higher dissolution rate of BG.^31^ This showed a good agreement with our work that the B-doped 58S BG specimens present better bioactivity when compared to the un-doped one. Although the addition of B could accelerate the dissolution of the silicate glass structure, Deilmann *et al.*^34^ demonstrated that a collapse of the pore network and loss of internal surface area may be facilitated with increased B concentration, hence could retard the uptake of Ca and the formation of HA. This agrees with our quantitative results (*I*_1_/*I*_2_ ratio) derived from FT-IR spectra (Fig. 6), indicating that the order of bioactivity is 1 mol% B-doped 58S BG > 3 mol% B-doped 58S BG > 5 mol% B-doped 58S BG > un-doped 58S BG.

Lastly, based on the MTT assays shown in Fig. 7, the osteoblast and angiogenic activities were discussed. To begin with, for the osteoblast activity, all specimens show viability over 100% *versus* the control, indicating non-toxic and proliferation against the MC3T3-E1 osteoblast cells. Based on the previous study,^19^ HA formation could lead to a positive effect on cell viability. In addition, the un-doped 58S BG specimen passed the ISO standard threshold with cell viability of 85% for the angiogenic activity, showing non-toxicity to the BAOEC endothelial cells. Further, the addition of B showed significant increases in cell viability, suggesting that the addition of B is capable of stimulating the secretion of angiogenic growth factors.^16,17^ In summary, the results indicated that incorporating B in 58S BG specimens could facilitate osteoblast and angiogenic activity, showing a good agreement with previous works.^35^

## 5. Conclusions

In this work, the spray drying method was utilized effectively for fabricating the un-doped and B-doped 58S BG microspheres. Corresponding characterizations of phase composition, surface morphologies, elemental composition, and ion distribution were carried out with discussion of the formation mechanism. The results suggested that all spray-dried 58S BG specimens exhibit amorphous phases. Meanwhile, two surface morphologies, smoothed and concaved spheres, were observed along with homogeneous ion distributions. Further, the XRD and FTIR analyses verified the *in vitro* bioactivity of all BG microspheres, meanwhile demonstrating the positive effect of the B dopant. Finally, both osteoblast and angiogenic activities were evaluated *via* MTT assay, and the results suggested that the B dopant is capable of enhancing both osteoblast and angiogenic activities significantly when compared to the un-doped specimen. In summary, results from this work demonstrated that the considerable potential of spray-dried B-doped BG microspheres, positioning them as promising candidates for future applications in tissue engineering.

## Conflicts of interest

There are no conflicts to declare.

## Supplementary Material
